# Evaluation of acute and subacute toxicity of ethanolic extract and fraction of alkaloids from bark of *Aspidosperma nitidum* in mice

**DOI:** 10.1038/s41598-021-97637-1

**Published:** 2021-09-14

**Authors:** Heliton Patrick Cordovil Brígido, Everton Luiz Pompeu Varela, Antônio Rafael Quadros Gomes, Mirian Letícia Carmo Bastos, Andre de Oliveira Feitosa, Andrey Moacir do Rosário Marinho, Liliane Almeida Carneiro, Márlia Regina Coelho-Ferreira, Maria Fâni Dolabela, Sandro Percário

**Affiliations:** 1 Post-graduate Program in Pharmaceutical Innovation, Institute of Health Sciences, Federal University of Pará, Belém, PA Brazil; 2 Post-graduate Program in Biodiversity and Biotechnology (BIONORTE), Institute of Biological Sciences, Federal University of Pará, Belém, PA Brazil; 3 Oxidative Stress Research Laboratory, Institute of Biological Sciences, Federal University of Pará, Av. Augusto Corrêa, 01, Belém, PA 66075-110 Brazil; 4 Post-graduate Program in Chemistry, Institute of Exact and Natural Sciences, Federal University of Pará, Belém, PA Brazil; 5 National Primate Center, Evandro Chagas Institute, Ananindeua, PA Brazil; 6 Botany Coordination, Museu Paraense Emílio Goeldi, Ministério da Ciência, Tecnologia, Inovação e Comunicações, Belém, PA Brazil

**Keywords:** Drug discovery, Toxicology

## Abstract

This study investigated the acute and subacute toxicity of the ethanolic extract (EE) and alkaloid fraction (FA) from *A. nitidum*. The EE was obtained from trunk bark with ethanol, FA was obtained from the fractionation of EE. To test the acute toxicity, mice were divided into four groups, and the negative controls received water or aqueous solution of dimethyl sulfoxide, whereas the others received EE or FA (2000 mg/kg, orally, single dose). The same controls were used in the subacute trial. However, the animals were treated for 28 days, and the dose used was 1000 mg/kg per day of EE and FA. Daily clinical evaluations of the animals were performed. At the end of the experiment, hematological, biochemical, and histopathological assessments (liver, lung, heart, and kidney) were performed. In the acute and subacute toxicity studies, mice treated with EE and FA did not show any clinical changes, there were no changes in weight gain, hematological and biochemical parameters compared to the control groups (p > 0.05). In the histopathological examination, there was no abnormality in the organs of the treated animals. Therefore, EE and FA did not produce toxic effects in mice after acute and subacute treatment.

## Introduction

*Aspidosperma nitidum* Benth. Ex Müll. Arg (Apocynaceae), popularly known as Carapanaúba, is a plant found in the Brazilian Amazon^1^ and widely used in local medicine to treat febrile illnesses, malaria^2^, uterus and ovary inflammation, diabetes, cancer, contraception, stomach problems^3^, and rheumatism^4^. In addition, indigenous people use its latex to treat leprosy^3^.

**Figure 1﻿:**
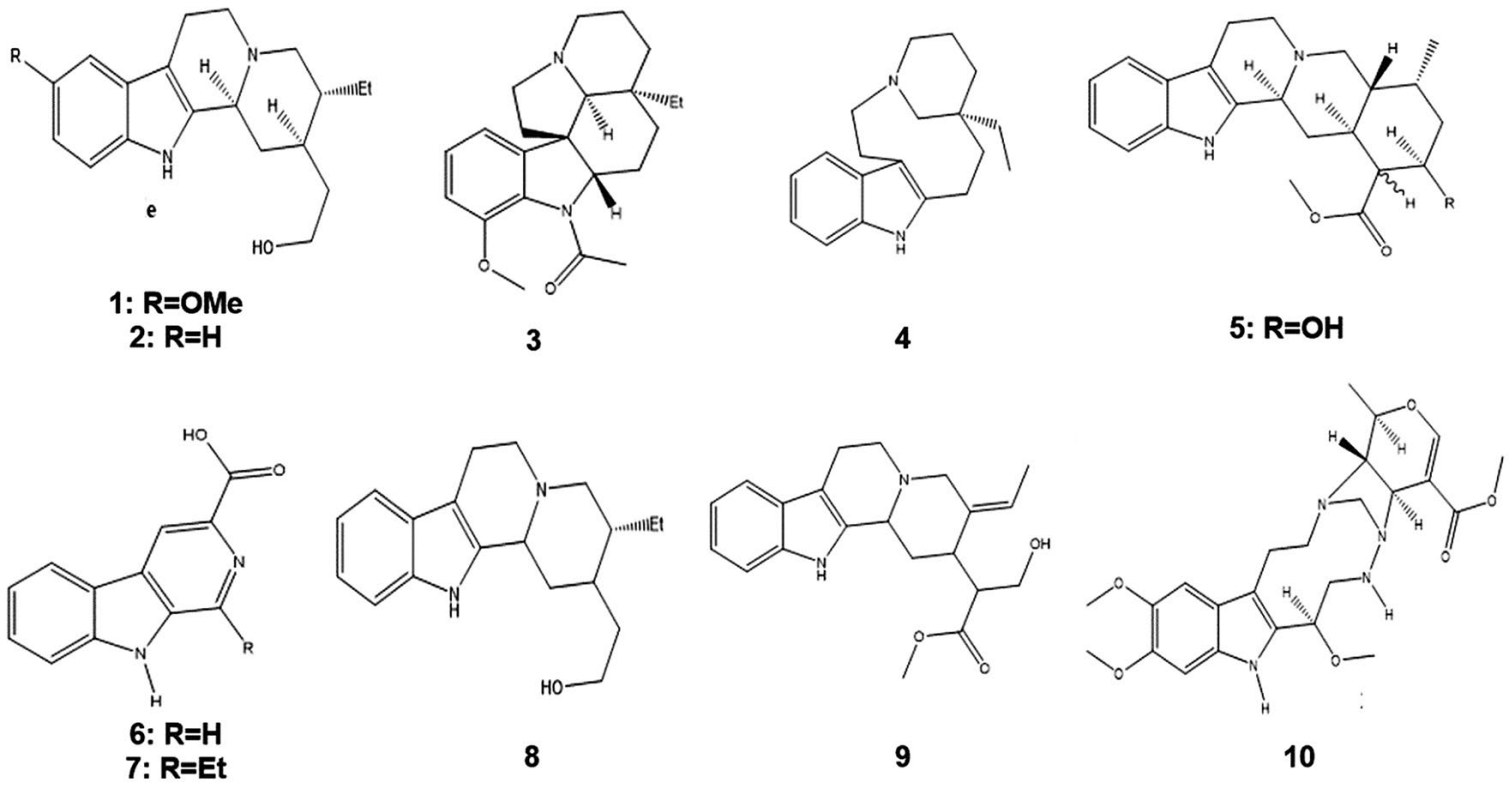
Chemical structure of compounds occurring in *Aspidosperma nitidum.* 10-methoxydihydrocorynantheol (1), corynantheol (2), aspidospermine (3), quebrachamine (4), yohimbine (5), carboxylic harman acid (6), 3-carboxylic ethylharman (7), dihydrocorynantheol (8), dehydrositsiriquine (9), braznitidumine (10).

**Figure 2:**
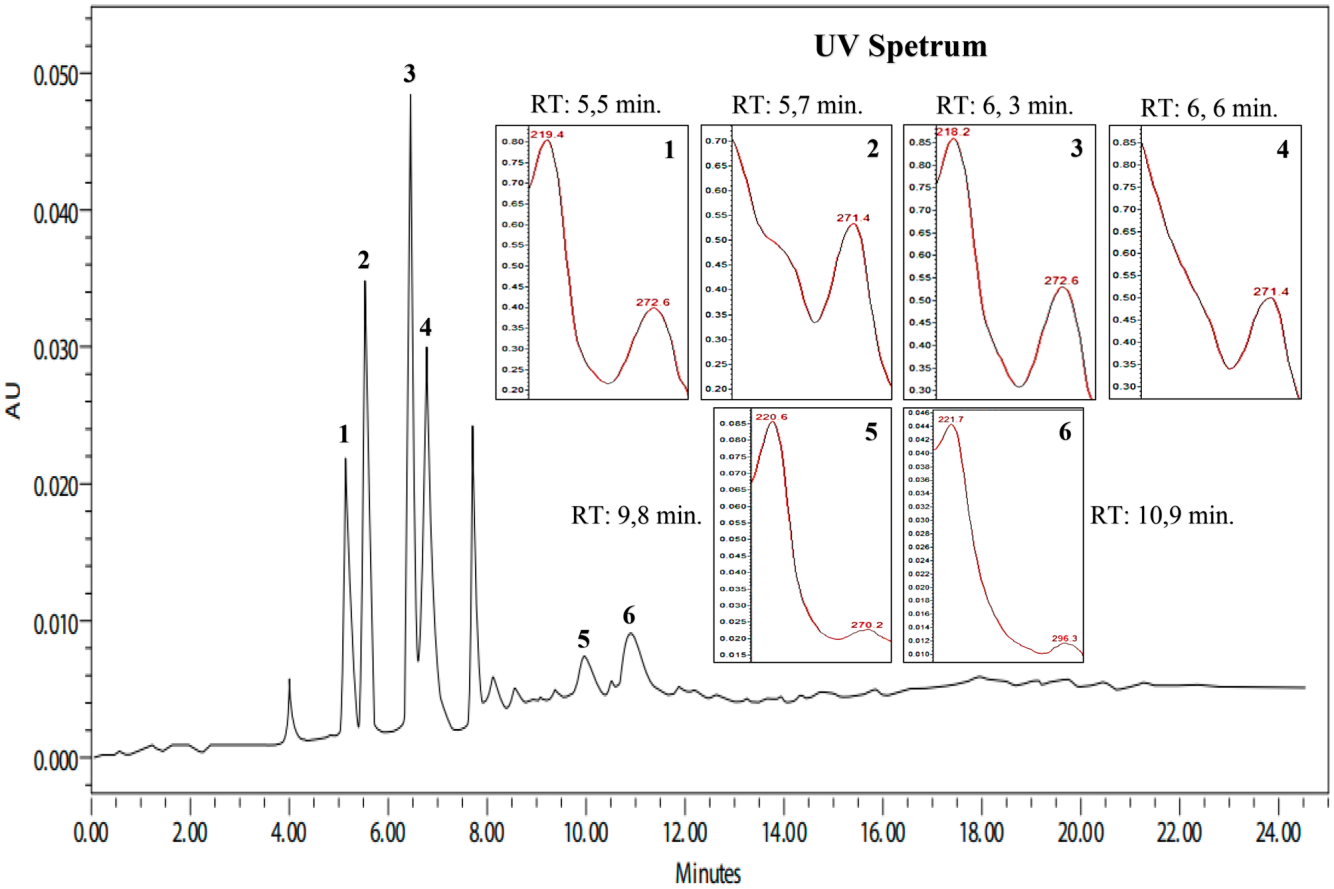
Chromatographic profile and UV spectra of the ethanolic extract of *Aspidosperma nitidum*. λ = 250–400 nm.

**Figure 3:**
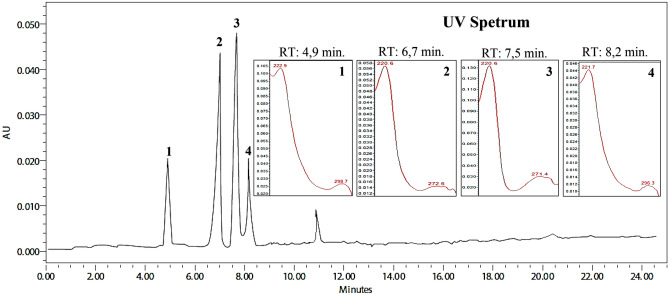
Chromatographic profile and UV spectra of the alkaloid fraction of *Aspidosperma nitidum*. λ = 250–400 nm.

**Figure 4:**
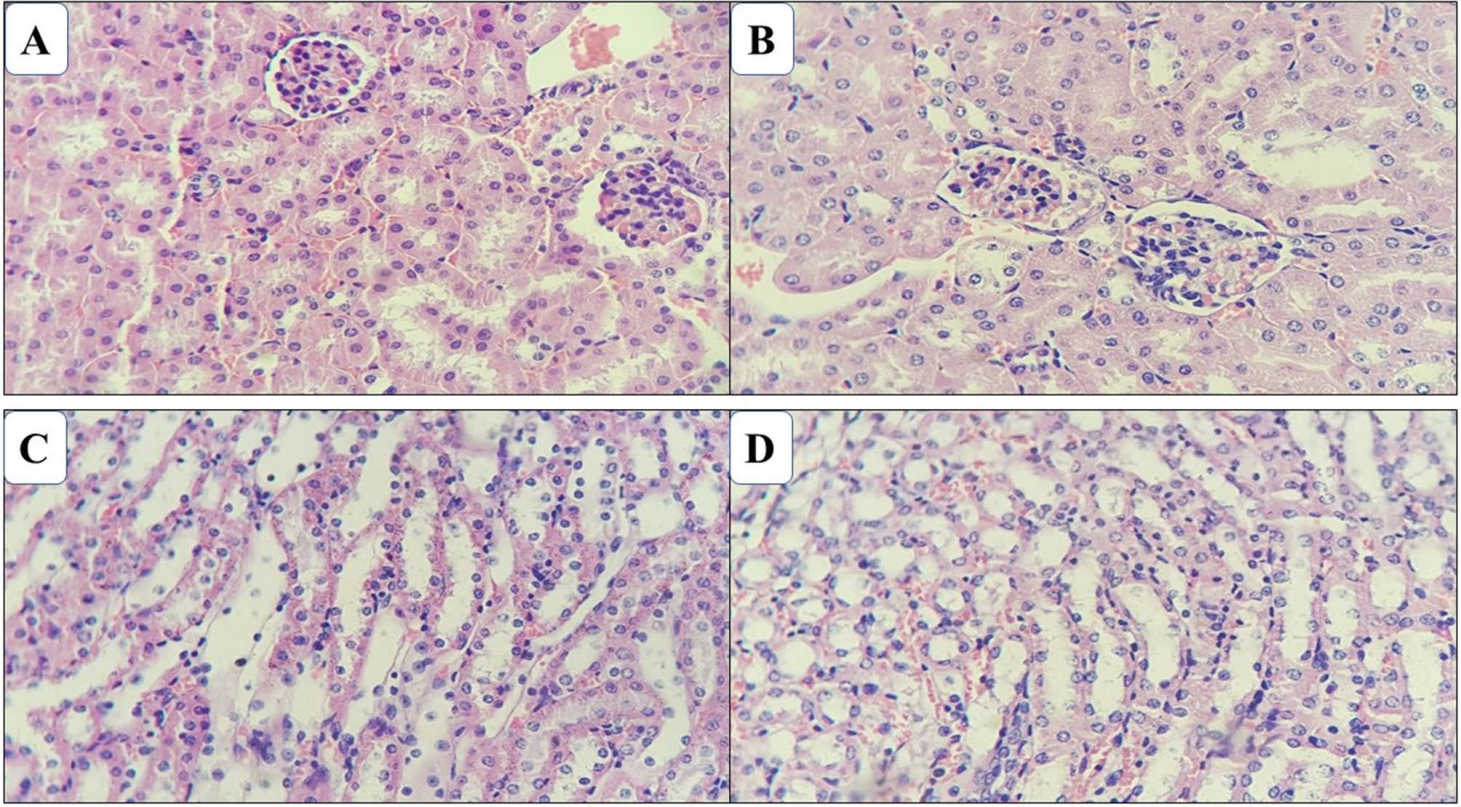
Photomicrograph of the kidneys of mice treated with ethanol extract and alkaloid fraction of *Aspidosperma nitidum*. (**A**) Group treated with ethanol extract; (**B**) control group (water:DMSO); (**C**) group treated with alkaloid fraction; (**D**) control group (water).

**Figure 5:**
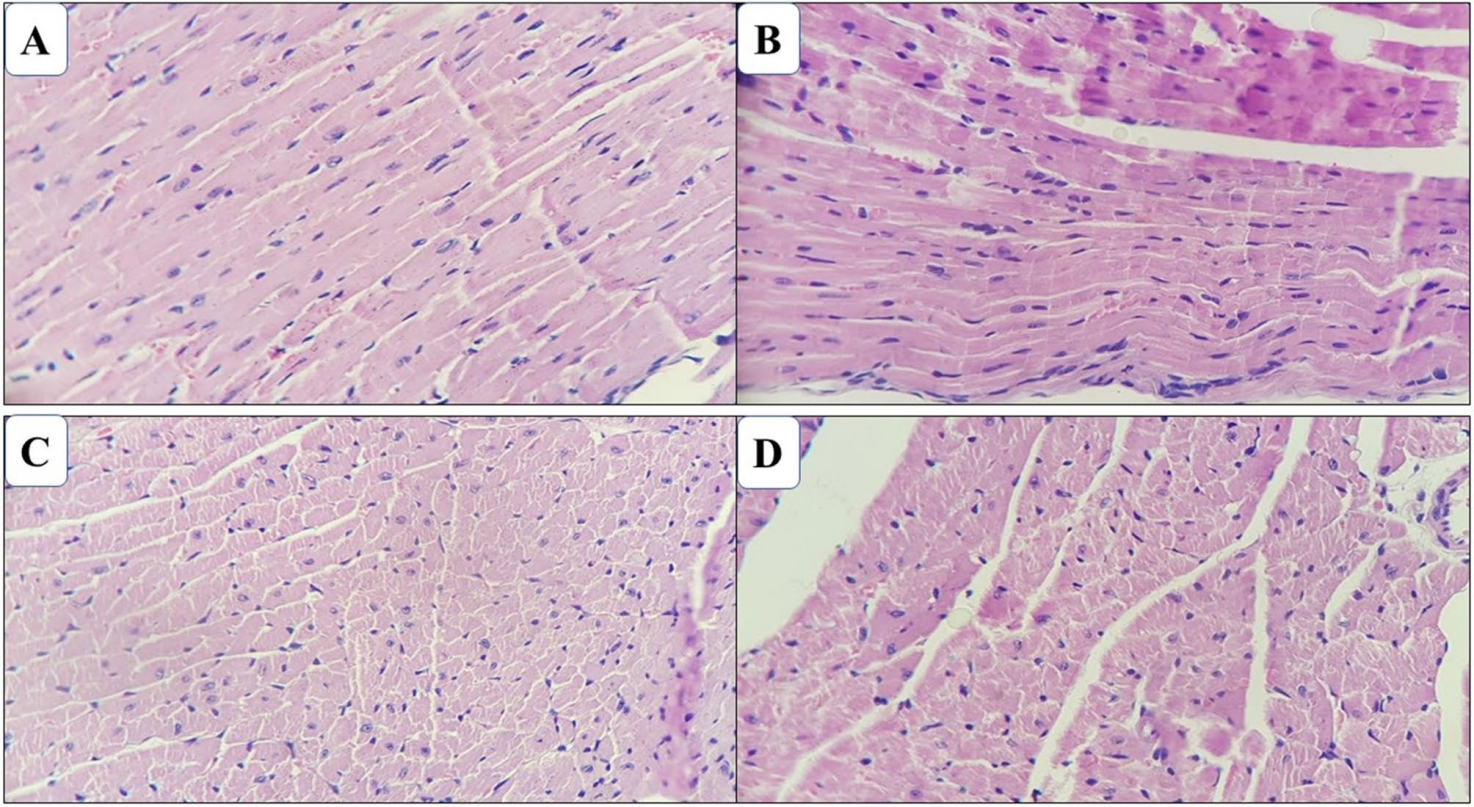
Photomicrograph of the heart of mice treated with ethanol extract and alkaloid fraction of *Aspidosperma nitidum*. (**A**) Group treated with ethanol extract; (**B**) control group (water:DMSO); (**C**) group treated with alkaloid fraction; (**D**) control group (water).

**Figure 6:**
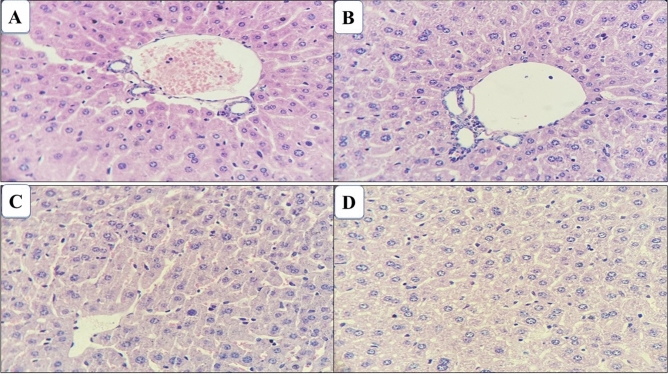
Photomicrograph of the liver of mice treated with ethanol extract and alkaloid fraction of *Aspidosperma nitidum*. (**A**) Group treated with ethanol extract; (**B**) control group (water:DMSO); (**C**) group treated with alkaloid fraction; (**D**) control group (water).

**Figure 7:**
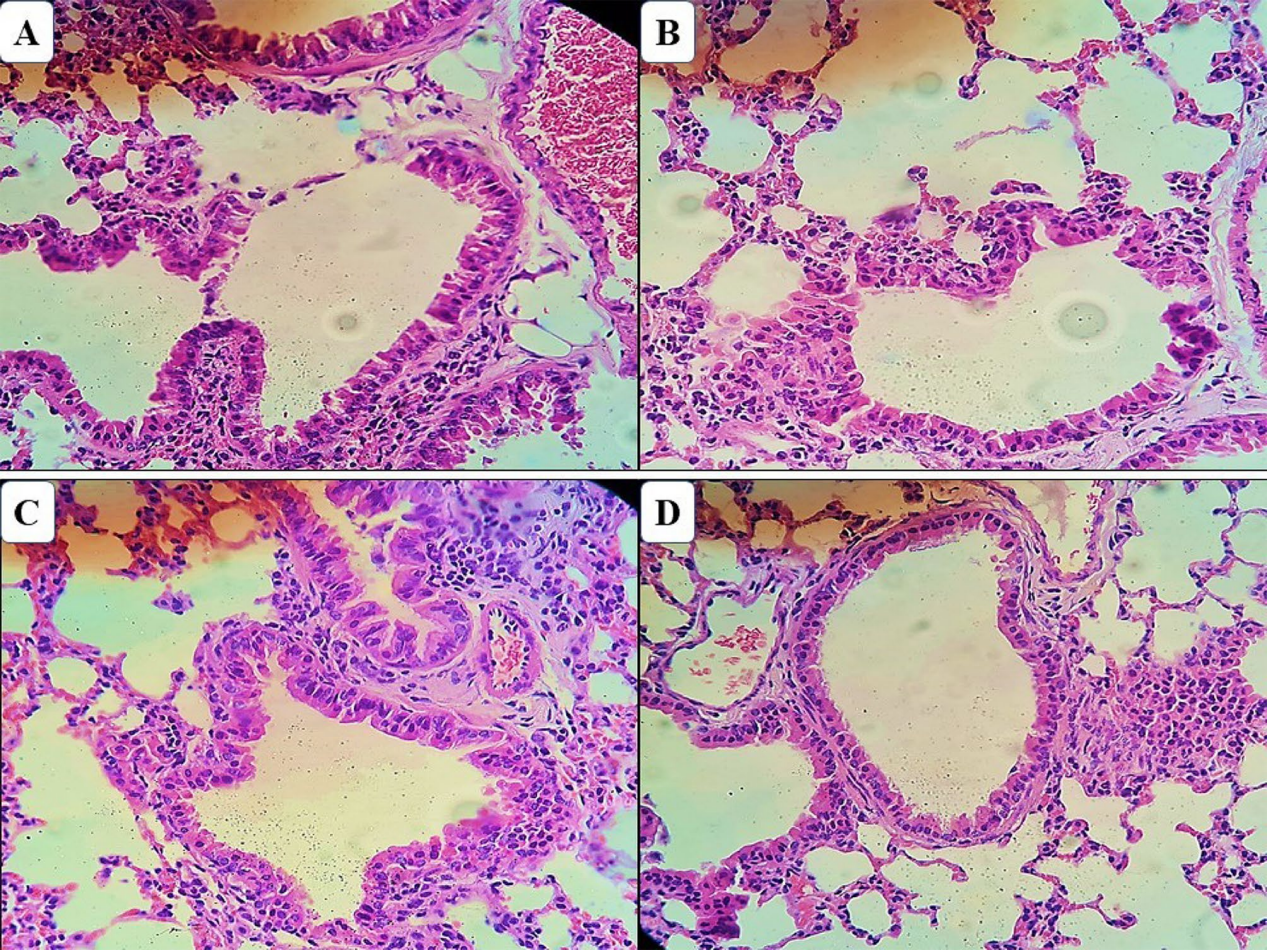
Photomicrograph of the lungs of mice treated with ethanol extract and alkaloid fraction of *Aspidosperma nitidum*. (**A**) Group treated with ethanol extract; (**B**) control group (water:DMSO); (**C**) group treated with alkaloid fraction; (**D**) control group (water).

Species belonging to the genus Aspidosperma are chemically characterized by the occurrence of indolic alkaloids^5–7^. In this context, the therapeutic properties of *A. nitidum* are mainly attributed to alkaloids^8^, with the following compounds having been isolated: 10-methoxydihydrocorynantheol (Fig. 1-1), corynantheol (Fig. 1-2)^9^, aspidospermine (Fig. 1-3), quebrachamine (Fig. 1-4), yohimbine (Fig. 1-5)^10^, carboxylic harman acid (Fig. 1-6), 3-carboxylic ethylharman (Fig. 1-7)^8^, dihydrocorynantheol (Fig. 1-8), dehydrositsiriquine (Fig. 1-9), and braznitidumine (Fig. 1-10)^11^.

A study demonstrated that the ethanolic extract from the stem bark of *A. nitidum* showed anti-inflammatory activity in the carrageenan induced rat paw edema method, with braznitidumine being responsible for this activity^12^.

Another study accessed the in vitro antimalarial activity of EE (IC_50_ = 3.6 µg/mL) and FA (IC_50_ = 2.32 µg/mL), both displaying low cytotoxicity for HepG2 cells (EE, IC_50_ = 410.65 µg/mL; FA, IC_50_ = 346.73 µg/mL), and highly selectivity for antimalarial activity (Selective Index—SI: EE = 114.07; FA = 149.45). The ethanolic extract and the alkaloid fraction reduced the parasitemia of mice infected with *Plasmodium berghei* (ANKA) by 80% (dose of 500 mg/kg) on the 5th day. In preliminary studies of acute oral toxicity, the ethanolic extract (5000 mg/kg; gavage) presented low toxicity, with no clinical or anatomopathological changes^13^.

Despite its wide use in popular medicine, its phytochemical diversity, and studies that validate its clinical use, toxicity studies are still scarce, and the results are, at most, preliminary. Thus, one important component of this study was the scientific assessment of the *A. nitidum* safety and toxicity.

This study result may fill the gap of previous studies about the species and provide some additional evidence to recommend further studies to assess the toxicity profiles associated with the use of herbal preparations from this plant as well. Thus, this study aimed to investigate the acute and subacute toxicity of the ethanolic extract (EE) and alkaloid fraction (FA) obtained from trunk bark of *A. nitidum*.

## Methods

### Plant material

Trunk barks of *A. nitidum* were collected on the state highway PA-150 (coordinates S 02° 09′ 50.3″ and W 048° 47′ 56.9″), in the state of Pará-Brazil, during August 2017. The plant material was identified by Dr. Márlia Regina Coelho-Ferreira and the exsiccate was deposited at the Herbarium João Murça Pires of the Museu Paraense Emílio Goeldi, under no. MG206608. In the present study, we used a wild plant collected from a virgin forest of the Amazon, and our work posed no risk of extinction for the species. During the collection, we took all care to remove the barks so as not to cause any damage to the species, in addition, only a small proportion of the barks were collected. The species were kept integrated and survived the collection. The project complies with national and international guidelines and legislation and is registered on the platform of the National Management and Genetic Heritage System and Associated Traditional Knowledge (SISGEN), whose provided license to collect the species under registration A2C3188. Moreover, according to the IUCN 2019 red list of endangered species, *Aspidosperma excelsum*, a synonymy of *Aspidosperma nitidum* is classified as Least Concern^14^.

### Preparation of extract and fraction of alkaloids

The barks of *A. nitidum* were washed under running water and dried in a circulating air oven (40 °C, for 7 days). The dry material was subjected to grinding in a knife mill. The plant powder was subjected to maceration with ethanol at 96°GL (1:10 ratio). The ethanolic solution was filtered and concentrated on a rotary evaporator under reduced pressure until total evaporation of the alcohol, yielding the dry ethanolic extract (EE). The EE (5 g) was subjected to acid–base partition, being solubilized in ethanol (4.0 mL), then 3% aqueous hydrochloric acid solution (7.5 mL) was added. This solution was extracted with dichloromethane (250 mL for three times), yielding the neutral fraction (FN). The acidic aqueous layer was made alkaline with 10% ammonium hydroxide (NH_4_OH) until pH 9, followed by a new extraction with dichloromethane (250 mL for three times), yielding an alkaline aqueous layer and an organic layer (fraction of alkaloids—FA).

### High performance liquid chromatography coupled to a diode array detector (HPLC–DAD)

The HPLC–DAD analyzes of the ethanolic extract and FA were carried out according to the adapted methodology of Coutinho et al.^15^. The extract and FA (1 mg) were solubilized in HPLC grade methanol (1 mL) under sonication (ultrasound) for 15 min. The column used was LiChrospher 100 RP-18 (particles of 5 mm, 250 × 4 mm *d.i.*), UV detection at 220–400 nm, flow of 0.5 mL/min, at 40 °C. Water (eluent A) and acetonitrile (eluent B) were used as the mobile phase. A linear gradient was used: 70–30% of eluent B for 15 min, 60–40% of eluent B for 20 min, 50–100% of eluent B for 25 min.

### Animals

Thirty-two (thirty-two) healthy Balb/c male mice (*Mus musculus*), adults, aged 6–8 weeks, weighing between 25 and 35 g, from the Vivarium of the Evandro Chagas Institute (Ananindeua-Pará, Brazil) were used. The animals were housed in the Experimental Vivarium of the Oxidative Stress Research Laboratory of ICB/UFPA, in polypropylene cages (30 × 19 × 13 cm), with a stainless-steel wire cover, containing a bed of Pine shaving, with a maximum of five animals per cage and kept at room temperature (24 ± 2 °C) and light/dark cycle every 12 h. Before and during the study period, the animals were kept with food (Presence, São Paulo-SP, Brazil) and water ad libitum. Before any experimental procedure, the animals were acclimated to laboratory conditions for 15 days. The experimental procedures with mice were performed at the Oxidative Stress Research Laboratory (LAPEO/ICB/UFPA) and were performed according to the ethical standards of animal experimentation indicated by the Brazilian Society of Laboratory Animal Science (SBCAL) and international standards^16^.

While under the effects of the anesthesia (9 mg/kg of ketamine 10% and 10 mg/kg of xylazine 2%), animals underwent euthanasia through hypovolemia induction, after the collection of the total volume of blood available from each animal by cardiac puncture.

### Ethics declaration

All animal procedures were strictly in accordance with the National Institutes Guide for the Care and Use of Laboratory Animals^16^ and approved by the Animal Use Ethics Committee of the Evandro Chagas Institute (CEUA-IEC), under the number 38/2017. Furthermore, this study was conducted according to ARRIVE guidelines^17^.

### Experimental procedures

#### Acute toxicity assessment

The acute oral toxicity test was performed according to the experimental protocol *Guideline* 423 of the *Organization for Economic Cooperation and Development* (OECD)^18^, with an initial dose of 2000 mg/kg of EE or FA. The number of animals used in this evaluation followed OECD (2001) guidance and reduction principle^19^, i.e., using the lesser possible number of animals to obtain statistical relevance.

The animals were randomly divided into four groups (n = 3). The first group received orally a single dose of EE (2000 mg/kg) dissolved in water. The second group received orally a single dose of FA (2000 mg/kg) dissolved in an aqueous solution containing 99:1 (v/v) dimethyl sulfoxide. The last two groups (control groups) received water (third group) and aqueous solution containing 99:1 (v/v) dimethyl sulfoxide (fourth group).

The following parameters were observed during the test; general activity, vocal frantic, irritability, touch response, tail grip response, contortion, posterior train position, straightening reflex, body tone, force to grasp, ataxia, auricular reflex, corneal reflex, tremors, convulsions, anesthesia, lacrimation, ptosis, urination, defecation, piloerection, hypothermia, breathing, cyanosis, hyperemia, and death.

#### Subacute toxicity assessment

For the subacute toxicity assessment of repeated doses of EE and FA, the methodology described in Guide 407 of the OECD guidelines^20^ and Brito^21^ was used, using the limit test with a dose of 1000 mg/kg of EE or FA.

The animals were randomly divided into four groups (n = 5). The first group received an oral dose of EE (1000 mg/kg) dissolved in water for 28 days. The second group received orally a daily dose of FA (1000 mg/kg) dissolved in an aqueous solution containing 99:1 (v/v) dimethyl sulfoxide for 28 days. The last two groups (control groups) received daily water (third group) and aqueous solution containing 99:1 (v/v) dimethyl sulfoxide (fourth group) for 28 days. The animals were observed daily during the experiment to detect death or abnormal clinical signs.

#### Observational parameters

After sample administration, the animals were kept under close observation continuously for 1 h and intermittently for the next 4 h and, thereafter, once every 12 h for the next 14 days for the acute toxicity assessment, and for 28 days for the subacute toxicity study.

Throughout the study period, clinical observations were made for mortality, behavioral, neurological, or other abnormalities, and their weight was measured weekly until the last day of experimentation.

The animals were evaluated at 30 min, 1 h, 2 h, 4 h, 6 h, 12 h, and 24 h and, from then on, daily, until the 14th day after treatment. The following signs were evaluated following Hippocratic screening: general activity, vocal frantic, irritability, touch response, tail grip response, contortion, posterior train position, straightening reflex, body tone, force to grasp, ataxia, auricular reflex, corneal reflex, tremors, convulsions, anesthesia, lacrimation, ptosis, urination, defecation, piloerection, hypothermia, breathing, cyanosis, hyperemia, and death. The signs evaluated by behavioral observation and systematic clinical examination of the animals were recorded in a printed protocol with the list of signs to be investigated^22,23^.

#### Hematological parameters

At the end of the experiment, animals were anesthetized (9 mg/kg of ketamine 10% and 10 mg/kg of xylazine 2%), and blood samples were drawn from each animal by cardiac puncture. The blood was placed in two groups of test tubes, half of the tubes containing the anticoagulant ethylenediamine tetra acetic acid (EDTA) and the other half without anticoagulant.

Blood samples in test tubes containing EDTA were used to determine hematological parameters: white blood cell count (WBC), red blood cell count (RBC), hemoglobin (HGB), hematocrit (HCT), mean cell volume (MCV), mean corpuscular hemoglobin concentration (MCHC), mean corpuscular hemoglobin (MCH), and platelet count (PLT), determined by the automatic method using the BC-2800 VET/Mindray device (Mindray do Brasil Ltda.; São Paulo, SP-Brazil). Differential counting was performed using a smear of blood stained by the panoptic.

Blood samples in test tubes without anticoagulant were left at room temperature to clot, and serum was obtained by centrifugation (3000 rpm for 10 min). Subsequently, biochemical analyzes were performed on serum to quantify aspartate aminotransferase (AST) and alanine aminotransferase (ALT) to assess liver damage and creatinine and urea to assess kidney damage, using an automated biochemical system. Standard commercial reagents (Labtest^®^, Labtest Diagnóstica SA, Lagoa Santa-MG, Brazil) were used, with kinetic, enzymatic, or colorimetric methods, at 37 °C, and the reading was performed on a semi-automatic spectrophotometer (Bio-Plus^®^ Biochemical Analyzer; Bioplus Produtos para Laboratórios Ltda.; Barueri-SP, Brazil).

#### Histopathological parameters

After euthanasia, liver, kidney, lung, and heart samples (1 cm thick) were collected for histopathological examination^21^. The heart, liver, and kidneys were sectioned by sagittal incision. The tissue sections were fixed in buffered formalin (10% formaldehyde) and after 24 h, cleaved for histopathological processing: dehydration with increasing series of alcohol (70°–100°), followed by diaphanization in xylol, impregnation and inclusion in paraffin, according to the usual methods^24^. In a microtome, tissue fragments (3.0 µm) were sectioned with subsequent hematoxylin–eosin staining for microscopic examination (40× and 100×). Slides were evaluated by an independent certified histopathologist, and the results were confirmed by a second independent certified histopathologist.

### Statistical analysis

The results obtained in each experiment were compared to its matched control group (i.e., EE-treated animals versus water-treated animals and FA-treated animals versus DMSO/water-treated animals) by Student's T-test using Excel program, with a 95% confidence level and a significance level of α = 5% (p < 0.05). The variables analyzed were expressed as mean ± standard deviation.

## Results

### HPLC–DAD analysis of *A. nitidum* extract and fractions

The EE chromatogram showed substances of high, medium, and low polarity. The main peaks of 5.5 min (λ_max_ 219.4, 272.6, and 364.1 nm), 5.7 min (λ_max_ 219.0; 271.4 and 358.2 nm), 6.3 min (λ_max_ 218.2; 272.6 and 376.1 nm), and 6.6 min (λ_max_ 271.4 and 357.2) showed ultraviolet spectra. The peaks with retention times of 9.8 min (λ_max_ 221.8 and 272.5 nm) and 10.9 min (λ_max_ 221.7 and 296.3 nm) showed absorbance (Fig. 2).

The FA chromatogram suggests that the main peaks identified at times of 4.9 min (λ_max_ 222.9 and 298.7 nm), 6.7 min (λ_max_ 220.6 and 272.6 nm), 7.5 min (220_max_ 220.6 and 271.4 nm), 8.2 min (λ_max_ 221.7 and 296.3 nm; Fig. 3).

### Acute toxicity assessment

The EE and FA orally administered at a dose of 2000 mg/kg in male mice did not induce any death or toxic symptoms in treated mice. All animals displayed normal behavior throughout the study and survived until the end of the 14-day experiment period. During the entire observation period, they did not present any significant clinical alteration. Furthermore, no significant difference was observed between the weight gain in these groups in relation to the controls (Table 1). Hematological analyzes showed that EE and FA did not cause significant changes of any of the parameters (Tables 2 and 3) and did not alter renal or hepatic function (Table 4), remaining within the reference range. As a result, the LD_50_ of EE and FA was greater than 2000 mg/kg of body weight.

**Table 1:** Weight of mice treated with ethanolic extract or fraction of alkaloids from *Aspidosperma nitidum.*

Treatment duration (days)	Weight (g)		p*
	EE	WCG	
0	27.06 ± 0.75	26.63 ± 0.20	0.183
7	27.9 ± 1.15	27.73 ± 0.11	0.742
14	27.26 ± 1.20	28.63 ± 0.15	0.225

	FA	DCG	(p)
0	26.23 ± 0.25	26.86 ± 0.58	0.183
7	27.06 ± 0.05	27.86 ± 0.35	0.423
14	28.1 ± 0.20	28.90 ± 0.17	0.230

*EE* ethanolic extract, *FA* fraction of alkaloids, *WCG* water control group, *DCG* DMSO control group (99% water + 1% DMSO).

*p value was obtained by Student's T-test, comparing EE versus WCG, or FA versus DCG.

**Table 2:** Erythrogram and platelet count of mice treated with ethanolic extract or alkaloid fraction from *Aspidosperma nitidum.*

Parameters	Groups		p*	RV^25^	
	EE	WCG		LABC	CPqRR
Red blood cells (10^6^/mm3)	6.60 ± 0.44	6.14 ± 1.13	0.251	7.4–11.1	5.8–10.5
Hematocrit (%)	35.36 ± 2.54	37.9 ± 0.56	0.647	34.3–51.3	28.3–68.3
Hemoglobin (g/dL)	13.1 ± 0.36	11.46 ± 2.72	0.346	11.5–15.9	10.1–18.4
MCV	53.5 ± 1.74	52.7 ± 0.49	0.851	46.8–48.0	47.0–51.0
MCH	19.86 ± 0.76	18.5 ± 1.58	0.275	14.1–16.9	14.6–18.5
MCHC	37.1 ± 1.57	35.3 ± 2.66	0.398	30.5–35.4	32.4–37.2
Platelet (10^3^/mm^3^)	1329.3 ± 29.02	1342.5 ± 72.8	0.439	635–1118	179–1025

	FA	DCG	P*	LABC	CPqRR
Red blood cells (10^6^/mm^3^)	6.71 ± 1.38	6.77 ± 0.06	1.000	7.4–11.1	5.8–10.5
Hematocrit (%)	35.33 ± 9.84	37.93 ± 0.75	0.716	34.3–51.3	28.3–68.3
Hemoglobin (g/dL)	12.66 ± 2.40	12.8 ± 0.3	1.000	11.5–15.9	10.1–18.4
MCV	52.1 ± 4.01	55.7 ± 0.79	0.232	46.8–48.0	47.0–51.0
MCH	18.93 ± 2.07	18.5 ± 1.58	0.692	14.1–16.9	14.6–18.5
MCHC	36.4 ± 4.10	35.3 ± 2.66	0.763	30.5–35.4	32.4–37.2
Platelet (10^3^/mm^3^)	1561 ± 42.4	1336.6 ± 52.4	0.704	635–1118	179–1025

*EE* ethanolic extract, *FA* fraction of alkaloids, *WCG* water control group, *DCG* DMSO control group (99% water + 1% DMSO), *RV* reference value, *LABC* Laboratory Animal Breeding Center, *CPqRR* René Rachou Research Center.

*p value was obtained by Student's T-test, comparing EE versus WCG, or FA versus DCG.

**Table 3:** Leukogram of mice treated with ethanolic extract or alkaloid fraction from *Aspidosperma nitidum.*

Parameters	Groups		p*	RV^25^	
	EE	WCG		LABC	CPqRR
Leukocytes (mm^3^)	2269.3 ± 0.07	2400 ± 0.15	0.423	2000–5900	1600–4100
Lymphocyte (%)	79.05 ± 12.65	76.66 ± 9.06	0.793	56–92	62.0–98
Lymphocyte (µL)	1801.8 ± 0.14	1860 ± 0.14	1.000	1280–4956	1050–3360
Neutrophil (%)	13.65 ± 11.24	15.46 ± 5.48	0.714	8.0–32.0	2.0–36
Neutrophil (µL)	300.3 ± 0.11	330 ± 0.03	0.635	288–1248	34.0–1050
Monocyte (%)	0.33 ± 0.57	0.06 ± 0.11	–	0.0–6.0	0.0–4.0
Monocyte (µL)	7.4 ± 0.87	1.44 ± 0.0	–	0.0–168	0.0–160
Eosinophil (%)	7.3 ± 1.41	7.8 ± 7.80	0.561	0.0–4.0	0.0–2.0
Eosinophil (µL)	150 ± 0.43	190.8 ± 0.07	0.633	0.0–96.0	0.0–44

	FA	DCG	p*	LABC	CPqRR
Leukocytes (mm^3^)	3330 ± 0.42	2402.4 ± 0.06	0.021	2000–5900	1600–4100
Lymphocyte (%)	84.4 ± 2.36	74.46 ± 6.30	0.003	56–92	62.0–98
Lymphocyte (µL)	2820 ± 0.32	1920 ± 0.09	0.037	1280–4956	1050–3360
Neutrophil (%)	10.63 ± 2.25	15.5 ± 5.38	0.014	8.0–32.0	2.0–36
Neutrophil (µL)	360 ± 0.54	42 ± 0.42	0.023	288–1248	34.0–1050
Monocyte (%)	0.03 ± 0.05	0.1 ± 0.1	–	0.0–6.0	0.0–4.0
Monocyte (µL)	1.22 ± 0.34	2.4 ± 0.25	–	0.0–168	0.0–160
Eosinophil (%)	4.83 ± 2.85	7.86 ± 7.82	0.259	0.0–4.0	0.0–2.0
Eosinophil (µL)	165.5 ± 0.05	188.8 ± 0.09	0.139	0.0–96.0	0.0–44

*EE* ethanolic extract, *FA* fraction of alkaloids, *WCG* water control group, *DCG* DMSO control group (99% water + 1% DMSO), *RV* reference value, *LABC* Laboratory Animal Breeding Center, *CPqRR* René Rachou Research Center.

*p value was obtained by Student's T-test, comparing EE versus WCG, or FA versus DCG.

**Table 4:** Biochemical parameters of mice treated orally with a single dose of ethanolic extract or alkaloid fraction from *Aspidosperma nitidum.*

Parameters	Groups		P*	RV^25^	
	EE	WCG		LABC	CPqRR
AST (U/L)	88 ± 4.35	77.33 ± 3.05	0.203	64–258	175–193
ALT (U/L)	44.66 ± 3.21	40 ± 3.46	0.034	75–193	32–178
UREA (mg/dl)	45.53 ± 4.29	49.26 ± 4.14	0.345	22–51	27–70
CREATININE mg/dl)	0.24 ± 0.06	0.31 ± 0.03	0.207	0.2–0.6	0.2–0.9

	FA	DCG	p*	LABC	CPqRR
AST (U/L)	90 ± 7.93	83 ± 8.54	0.357	64–258	175–193
ALT (U/L)	86.66 ± 7.23	39.33 ± 4.72	0.000	75–193	32–178
UREA (mg/dl)	43.86 ± 1.92	49.4 ± 2.66	0.043	22–51	27–70
CREATININE (mg/dl)	0.27 ± 0.06	0.32 ± 0.04	0.378	0.2–0.6	0.2–0.9

*EE* ethanolic extract, *FA* fraction of alkaloids, *WCG* water control group, *DCG* DMSO control group (99% water + 1% DMSO), *AST* aspartate aminotransferase, *ALT* alanine aminotransferase, *RV* reference value, *LABC* Laboratory Animal Breeding Center, *CPqRR* René Rachou Research Center.

*p value was obtained by Student's T-test, comparing EE versus WCG, or FA versus DCG.

### Subacute toxicity studies

The ethanolic extract and the fraction of alkaloids orally administered to male mice daily treated with 1000 mg/kg/28 days did not induce any death or toxic symptoms to the animals, whose behaved normally throughout the study and survived until the end of the experiment (28 days). For the duration of the experiment, no significant clinical changes were observed, and the weight gain was also similar to the controls (WCG and DCG; Table 5). There were no changes in the hematological parameters of the mice (Tables 6 and 7). Moreover, no significant changes were observed in renal and hepatic function tests (Table 8). These parameters were within the reference range.

**Table 5:** Weight of mice treated with repeated doses of ethanolic extract or alkaloid fraction from *Aspidosperma nitidum.*

Treatment duration (days)	Weight (g)		p*
	EE	WCG	
0	28.62 ± 1.46	28.92 ± 1.13	0.713
7	29.38 ± 1.35	29.35 ± 1.12	0.903
14	30.14 ± 1.001	30.12 ± 1.41	0.713
28	30.96 ± 0.82	30.92 ± 1.51	0.806

	FA	DCG	p*
0	28.27 ± 1.60	28.48 ± 1.42	0.903
7	29.45 ± 1.39	29.08 ± 1.22	0.807
14	30.2 ± 1.16	29.84 ± 1.20	0.540
28	30.95 ± 1.09	30.62 ± 1.31	0.540

*EE* ethanolic extract, *FA* fraction of alkaloids, *WCG* water control group, *DCG* DMSO control group (99% water + 1% DMSO).

*p value was obtained by Student's T-test, comparing EE versus WCG, or FA versus DCG.

**Table 6:** Erythrogram and platelet count of mice treated with repeated doses of ethanolic extract or alkaloid fraction from *Aspidosperma nitidum.*

Parameters	Groups		p*	VR^25^	
	EE	WCG		LABC	CPqRR
Red blood cells (10^6^/mm^3^)	8.56 ± 0.30	8.25 ± 0.20	0.391	7.4–11.1	5.8–10.5
Hematocrit (%)	39.27 ± 1.08	38.07 ± 0.61	0.462	34.3–51.3	28.3–68.3
Hemoglobin (g/dL)	14.12 ± 0.35	13.75 ± 0.33	0.624	11.5–15.9	10.1–18.4
MCV	46.15 ± 0.49	46.1 ± 0.54	0.462	46.8–48.0	47.0–51.0
MCH	16.5 ± 0.22	16.67 ± 0.22	0.270	14.1–16.9	14.6–18.5
MCHC	35.92 ± 0.35	36.15 ± 0.23	0.111	30.5–35.4	32.4–37.2
Platelet (10^3^/mm^3^)	883.66 ± 24.13	765 ± 39.59	1.000	635–1118	179–1025

	FA	DCG	p*	LABC	CPqRR
Red blood cells (10^6^/mm^3^)	8.40 ± 0.19	8.64 ± 0.16	0.083	7.4–11.1	5.8–10.5
Hematocrit (%)	38.66 ± 0.37	38.4 ± 0.80	0.564	34.3–51.3	28.3–68.3
Hemoglobin (g/dL)	13.83 ± 0.15	13.6 ± 0.81	0.773	11.5–15.9	10.1–18.4
MCV	46.03 ± 0.45	45.9 ± 0.43	0.564	46.8–48.0	47.0–51.0
MCH	16.57 ± 0.40	16.75 ± 0.68	0.885	14.1–16.9	14.6–18.5
MCHC	35.53 ± 0.83	36.37 ± 0.85	0.061	30.5–35.4	32.4–37.2
Platelet (10^3^/mm^3^)	849 ± 12.72	730 ± 36.42	0.563	635–1118	179–1025

*EE* ethanolic extract, *FA* fraction of alkaloids, *WCG* water control group, *DCG* DMSO control group (99% water + 1% DMSO), *RV* reference value, *LABC* Laboratory Animal Breeding Center, *CPqRR* René Rachou Research Center.

*p value was obtained by Student's T-test, comparing EE versus WCG, or FA versus DCG.

**Table 7:** Leukogram in mice treated with repeated doses of ethanolic extract or alkaloid fraction from *Aspidosperma nitidum.*

Parameters	Groups		p*	RV^25^	
	EE	WCG		LABC	CPqRR
Leukocytes (mm^3^)	2204 ± 515.9	2222 ± 632.75	0.711	2278–5900	1600–4100
Lymphocyte (%)	96.54 ± 2.83	96.07 ± 2.48	0.540	56–92	62.0–98
Lymphocyte (µL)	2126 ± 538.4	2137 ± 619.9	0.807	1280–4956	1050–3360
Neutrophil (%)	2.14 ± 1.82	2.25 ± 1.56	0.713	8.0–32.0	2.0–36
Neutrophil (µL)	42 ± 33.46	50.17 ± 35.39	0.807	288–1248	34.0–1050
Monocyte (%)	0.0 ± 0.0	0.0 ± 0.0	–	0.0–6.0	0.0–4
Monocyte (µL)	0.0 ± 0.0	0.0 ± 0.0	–	0.0–168	0.0–160
Eosinophil (%)	1.32 ± 1.01	1.67 ± 1.44	0.540	0.0–4.0	0.0–2.0
Eosinophil (µL)	18.5 ± 17.38	37.37 ± 33.79	0.602	0.0–96.0	0.0–44

	FA	DCG	p*	LABC	CPqRR
Leukocytes (mm^3^)	2100 ± 234.9	2278 ± 561.7	1.000	2278–5900	1600–4100
Lymphocyte (%)	96.2 ± 2.54	95.66 ± 2.34	0.563	56–92	62.0–98
Lymphocyte (µL)	2027 ± 276.5	2180 ± 545.2	1.000	1280–4956	1050–3360
Neutrophil (%)	2.25 ± 1.50	2.5 ± 1.46	0.773	8.0–32.0	2.0–36
Neutrophil (µL)	45.25 ± 29.15	57.64 ± 34.9	1.000	288–1248	34.0–1050
Monocyte (%)	0.0 ± 0.0	0.2 ± 0.44	–	0.0–6.0	0.0–4
Monocyte (µL)	0.0 ± 0.0	5.0 ± 11.18	–	0.0–168	0.0–160
Eosinophil (%)	1.55 ± 1.16	1.74 ± 1.25	0.563	0.0–4.0	0.0–2.0
Eosinophil (µL)	27.5 ± 17.07	39.89 ± 29.8	0.194	0.0–96.0	0.0–44

*EE* ethanolic extract, *FA* fraction of alkaloids, *WCG* water control group, *DCG* DMSO control group (99% water + 1% DMSO), *RV* reference value, *LABC* Laboratory Animal Breeding Center, *CPqRR* René Rachou Research Center.

*p value was obtained by Student's T-test, comparing EE versus WCG, or FA versus DCG.

**Table 8:** Biochemical parameters of mice treated orally with ethanolic extract or alkaloid fraction from *Aspidosperma nitidum* for 28 days.

Parameters	Groups		p*	RV^25^	
	EE	WCG		LABC	CPqRR
AST (U/L)	76.6 ± 31.58	77.5 ± 2.06	0.624	64–258	175–193
ALT (U/L)	63.6 ± 39.84	42.5 ± 10.96	0.327	75–193	32–178
UREA (mg/dL)	51.68 ± 7.04	49.6 ± 3.02	1.000	22–51	27–70
Creatinine (mg/dL)	0.27 ± 0.02	0.32 ± 0.02	0.177	0.2–0.6	0.2–0.9

	FA	DCG	p*	LABC	CPqRR
AST (U/L)	89.0 ± 10.63	80 ± 7.52	0.248	64–258	175–193
ALT (U/L)	65.75 ± 28.53	44 ± 3.74	0.773	75–193	32–178
UREA (mg/dL)	47.77 ± 4.37	49.3 ± 2.70	0.773	22–51	27–70
Creatinine (mg/dL)	0.32 ± 0.03	0.35 ± 0.29	0.563	0.2–0.6	0.2–0.9

*EE* ethanolic extract, *FA* fraction of alkaloids, *WCG* water control group, *DCG* DMSO control group (99% water + 1% DMSO), *AST* aspartate aminotransferase, *ALT* alanine aminotransferase, *RV* reference value, *LABC* Laboratory Animal Breeding Center, *CPqRR* René Rachou Research Center.

*p value was obtained by Student's T-test, comparing EE versus WCG, or FA versus DCG.

Histopathological examination of the viscera of the animals surviving the oral treatment of repeated doses of EE and AF obtained from the bark of *A. nitidum* at a dose of 1000 mg/kg/28 days, did not detect relevant changes both in the animals of the control group and in those treated with the samples. In the kidneys, lobular architecture was preserved with medullary pyramids covered with cortical tissue. The cortex presented regularly distributed glomeruli, fine Bowman capsule. The proximal and distal contorted tubules and the segment of the collecting duct did not evidence histological particularities, as did the Henle loops and collecting ducts of the medullary pyramid. No inflammatory reaction was observed or fibrosis in the interstice (Fig. 4).

In the heart, the myocardium was represented by cardiac striated muscle cells, presenting transverse striations, and single or double nuclei, centrally positioned. The endocardium had endothelium supported by a thin basal membrane and coated cavities and heart valves. The epicardium, coated with mesothelial pavement cells, were fully preserved (Fig. 5).

The liver was observed that the hepatic parenchyma (consisting of the lobular center vein surrounded by hepatocytes cords and sinusoid capillaries) were regularly distributed and with preserved structures. The hepatocyte presented a polygonal shape with spherical nucleus and centralized fully preserved (Fig. 6).

Histopathological evaluation of lung tissue sections in mice of all test groups showed a normal morphological architecture without any pathological changes related to treatment and was similar to that of mice in the control group (Fig. 7).

## Discussion

In this study we sought to investigate the acute and subacute toxicity of the ethanolic extract (EE) and the fraction of alkaloids (FA) obtained from *A. nitidum,* and our results showed that both the extract and the FA did not present toxicity.

The phytochemical evaluation showed that EE and FA obtained from *A. nitidum* are basically constituted by alkaloids. In the EE chromatogram, the four main peaks (TR = 5.5 min, 5.7 min, 6.3 min, and 6.6 min) presented ultraviolet spectra suggestive of alkaloids, probably β-carbolinic alkaloids^26^. The peaks with retention times of 9.8 min and 10.9 min showed absorbance suggestive of chromophores of indole alkaloids^27^.

The FA chromatogram suggests that the main peaks identified at times of 4.9 min, 5.7 min, 7.5 min, and 8.2 min are suggestive of indole alkaloids with an aspidospermine nucleus^8^. It is worth mentioning that studies have demonstrated their antiplasmodial, antileishmanial, and antitrypanosomal activities^28^.

When comparing the chemical composition of EE in relation to FA, there was an absence of peaks suggestive of β-carbolinic alkaloids in FA. This fact can be explained by the method used for fractioning. The acid–base partition is more efficient for fractions containing indolic alkaloids, since β-carbolinic alkaloids, when in contact with hydrochloric acid, tend to form phenol-harmol pairs, which can be precipitated and retained in alkaline water solution^29^. Thus, only indole alkaloids, especially those containing the aspidospermine nucleus, are seen in FA chromatograms.

Regarding to the toxicity of traditional plants used to treat diseases, the World Health Organization recommends the development of scientific research on their toxic side effects^30^. Although *A. nitidum* has historically been used in folk medicine to treat and prevent various diseases such as fever and malaria^15,31^, to date, there are no reports on its toxicity assessment.

Toxicological studies are necessary to determine safety, demonstrating the need to assess the toxicological profile for the selection of a safe dose^32^. In this context, the acute and subacute oral toxicities of *A. nitidum* were investigated.

The acute toxicity test assesses the adverse effects that occur in a short time after the administration of a single high dose of a substance. This test is performed mainly on rodents and is usually done at the beginning of the development of a new substance to provide information about its potential toxicity^33^.

In this regard, the acute toxicity tests of EE and FA showed that, at the tested dose (2000 mg/kg), no toxic sign, behavioral change or death was observed. These results show that both the extract and the FA obtained from *A. nitidum* can be considered nontoxic based on the acute toxicity classification method^34^, with the LD_50_ of EE and FA greater than 2000 mg/kg of body weight.

Toxicological assessments after repeated dosing provide evidence of dose response with possible health risks after a 28-day subacute toxicity test. In the present study, a dose of 1000 mg/kg of EE or FA was administered for 28 days and no toxic effects, death, or abnormal signs, nor changes in weight were observed in mice treated with EE or FA. Such events indicate that these samples display no toxicity. Thus, EE and FA can be considered relatively safe for acute or subacute exposure.

Regarding the physiological and pathological state in humans and animals, hematopoietic parameters are considered the most sensitive markers to assess the toxic effects of substances^35^. It is known that variations in this system are a sensitive index for human toxicity if the data obtained in animal studies are transposed^36^. In the acute and subacute test of EE or FA, there was no noticeable change in the analyzed parameters, as well as in liver and kidney function assessment of the mice used in this study.

In this regard, some enzymes and proteins, including ALT and AST, are known as sensitive biomarkers of hepatocellular function^37^, being ALT considered a marker with higher sensibility and specificity for hepatotoxicity^38^, whereas AST responds very rapidly (24 h) to acute livre damage, with an increase up to ten-fold of baseline values^39,40^. When there is liver damage, the serum levels of AST and ALT increase^41^. On the other hand, renal function can be assessed by changes in urea nitrogen and creatinine, and an increase in these parameters indicates possible damage to renal function^42^. In this context, after treatment with EE or FA, plasma levels of AST, ALT, urea, and creatinine remained within physiological limits, suggesting that the acute and subacute administration of EE or FA does not interfere with hepatic metabolism, nor with renal excretion. Thus, it is safe to consider that both EE and FA did not induce harmful effects on kidneys and liver^43,44^.

Histopathological studies serve as supporting evidence for hematological and biochemical analyzes^45^. In the present study, the histological evaluation performed in the subacute test showed that animals treated with EE or FA did not show changes in color, shape, size, and texture of the liver, heart, lungs, and kidneys, when compared to matched control groups. These findings are in accordance with the observed hematological and biochemical parameters, suggesting that EE or FA do not cause any harmful effects on vital organs even when administered in repeated doses.

Other species of *Aspidosperma* have already been evaluated for toxicological potential. Gomes^46^, investigating the toxicity of a hydroethanolic extract obtained from the bark of *A. excelsum* against Swiss mice, used the dose of 5000 mg/kg orally administered and found that there were no deaths nor toxicity signs. It is worth remembering that *A. nitidum* is considered taxonomically synonymous with *A. excelsum*, and in this study a higher dose was used in relation to the present study and yet, considered nontoxic in the tested doses.

Another study by Carvalho^47^ evaluated the acute and subacute toxicity of the ethanolic extract obtained from the bark of *A. subincanum*, using a single dose of 300 mg/kg in Swiss mice (*M. musculus*). It showed no signs of toxicity, death, or behavioral changes in mice. In the subacute trial, the ethanolic extract was administered orally in doses of 75 mg/kg, 150 mg/kg, and 300 mg/kg, neither death nor any sign of toxicity was observed. According to the author, all results corroborate the hypothesis that extracts of *Aspidosperma* species have low toxic potential, similarly to the results obtained in our study.

In the extracts’ fractionation, there is the possibility of a higher concentration of a certain metabolite, according to the chemical characteristics of the substance to be fractionated. In this sense, changes in the activity and toxicity of substances can occur. In the present study, this fact was not observed since the FA did not show signs of acute and subacute toxicity. In other words, the fractionation carried out on *A. nitidum* did not influence the toxicity pattern of the fractions. Thus, the absence of toxicity from EE and FA suggests a possibility for the use of these plant extracts for a longer period of treatment.

In phytochemical terms, a great diversity of compounds has already been isolated from *A. nitidum*, among them, the indole alkaloids are the most identified. From the species, aspidospermine and yohimbine, which are widespread in other representatives of the genus, are mostly present in the barks, leaves, and branches^10^. Aspidospermine showed antimalarial activity against the chloroquine-resistant strain of *Plasmodium falciparum*^48^, and yohimbine acts as a blocker of α2-adrenergic and serotoninergic receptors, causing central excitation, elevated blood pressure, increased heart rate, and increased motor and antidiuretic activity^49^.

Nevertheless, there is still a lack of toxicity studies that evaluate the fractions of alkaloids obtained from extracts of *Aspidosperma* bark. As the different pharmacological activities of species belonging to this genus have been attributed to alkaloids, especially indole ones, it is important to assess whether obtaining fractions with higher levels of this metabolite contributes to increased toxicity. In vitro studies have shown that fractionation contributed to increased cytotoxicity in hepatoma cells (HepG2). The 50% cytotoxic concentration (CC_50_) of the extract was 410.65 + 9.84 µg/mL, and the alkaloid fraction was 346.73 + 14.17 µg/mL^50^. However, this change in toxicity was not observed in vivo.

Other studies have already evaluated the toxicity signs of extracts containing alkaloids belonging to species of the same family as *A. nitidum* (Apocynaceae). The species *Nerium oleander* (active substances: oleandrin, nerioside, and folineurin), and *Thevetia neriifolia*, in toxic doses, cause a clinical picture similar to digitalis, that is, neurological and cardiovascular disorders^51^. However, such signs were not observed in acute treatment or in repeated doses of the fraction of alkaloids from *A. nitidum.*

Studies involving species used in traditional medicine in Amazon are essential for analyzing the potential of the region's flora, as well as justifying safe use by natives. The results obtained in this study show *A. nitidum* is a promising plant, seen both by ethnobotanical studies and analysis of biological activity. Furthermore, our study did not demonstrate acute and subacute toxicity at the concentrations and duration of the tests, in addition, it is the first study that investigates the toxicity of *A. nitidum*.

## Conclusion

The study of acute and subacute toxicity of ethanolic extract and fraction of alkaloids obtained from *A. nitidum* was performed by oral administration using mice as an animal model. The results showed that both single-dose and repeated doses did not lead to mortality or signs of toxicity in mice. Therefore, the LD_50_ of the samples for mice is greater than 2000 mg/kg in the acute test and greater than 1000 mg/kg in the subacute test, suggesting a potential for safe use. Notwithstanding, further toxicological evaluations, including subchronic, chronic, and genotoxicity assessments are required to stablish its real safety.

## Data Availability

The datasets generated during and/or analyzed during the current study are available from the corresponding author on reasonable request.
